# Individually tailored dosage regimen of full-spectrum Cannabis extracts for autistic core and comorbid symptoms: a real-life report of multi-symptomatic benefits

**DOI:** 10.3389/fpsyt.2023.1210155

**Published:** 2023-08-21

**Authors:** Patrícia Soares Silva Montagner, Wesley Medeiros, Leandro Cruz Ramires da Silva, Clarissa Nogueira Borges, Joaquim Brasil-Neto, Vinícius de Deus Silva Barbosa, Fabio V. Caixeta, Renato Malcher-Lopes

**Affiliations:** ^1^NeuroVinci, São José, Brazil; ^2^Laboratory of Neuroscience and Behavior, Department of Physiological Sciences, Institute of Biological Sciences, University of Brasilia, Brasília, Brazil; ^3^Clinical Hospital, Federal University of Minas Gerais, Belo Horizonte, Brazil; ^4^Brazilian Association of Medical Cannabis Patients, Ama-Me, Belo Horizonte, Brazil; ^5^Specialized Educational Care Division for Gifted Students of the Department of Education of the Federal District, Brasília, Brazil; ^6^Euro-American University Center, Unieuro, Brasília, Brazil; ^7^Medical Cannabis Center–Syrian-Lebanese Hospital, São Paulo, Brazil; ^8^National Association for Inclusion of the Autistic People, São Paulo, Brazil

**Keywords:** endocannabinoid system, Pica, allotriophagy, *Cannabis sativa*, CBD, THC, patient-reported outcome survey, autism spectrum disorders

## Abstract

Autism Spectrum Disorders (ASD) may significantly impact the well-being of patients and their families. The therapeutic use of cannabis for ASD has gained interest due to its promising results and low side effects, but a consensus on treatment guidelines is lacking. In this study, we conducted a retrospective analysis of 20 patients with autistic symptoms who were treated with full-spectrum cannabis extracts (FCEs) in a response-based, individually-tailored dosage regimen. The daily dosage and relative proportions of cannabidiol (CBD) and tetrahydrocannabinol (THC) were adjusted based on treatment results following periodic clinical evaluation. Most patients (80%) were treated for a minimum of 6 months. We have used a novel, detailed online patient- or caregiver-reported outcome survey that inquired about core and comorbid symptoms, and quality of life. We also reviewed patients’ clinical files, and no individual condition within the autistic spectrum was excluded. This real-life approach enabled us to gain a clearer appraisal of the ample scope of benefits that FCEs can provide for ASD patients and their families. Eighteen patients started with a CBD-rich FCE titrating protocol, and in three of them, the CBD-rich (CBD-dominant) FCE was gradually complemented with low doses of a THC-rich (THC-dominant) FCE based on observed effects. Two other patients have used throughout treatment a blend of two FCEs, one CBD-rich and the other THC-rich. The outcomes were mainly positive for most symptoms, and only one patient from each of the two above-mentioned situations displayed important side effects one who has used only CBD-rich FCE throughout the treatment, and another who has used a blend of CBD-Rich and THC-rich FCEs. Therefore, after FCE treatment, 18 out of 20 patients showed improvement in most core and comorbid symptoms of autism, and in quality of life for patients and their families. For them, side effects were mild and infrequent. Additionally, we show, for the first time, that allotriophagy (Pica) can be treated by FCEs. Other medications were reduced or completely discontinued in most cases. Based on our findings, we propose guidelines for individually tailored dosage regimens that may be adapted to locally available qualified FCEs and guide further clinical trials.

## 1. Introduction

Autism spectrum disorders (ASD) are neurodevelopmental conditions characterized by two main symptom domains: (1) restricted or repetitive behaviors and (2) impairment in language/communication and social skills (1–11). ASD is commonly linked to a high burden of comorbid disorders. Symptoms presented by patients in the spectrum vary widely in severity, and may require either minor, substantial, or very substantial support for the patient’s everyday needs (5, 12). Besides the core symptomatic domains associated to ASD, people in the spectrum frequently present co-morbidities and neurodevelopmental disabilities. These include intellectual deficits (13, 14), behavioral problems (15), emotion regulation impairment (16), ingestion of nonfood substances (allotriophagy or PICA) (17–19), among others. All these conditions have considerable impact the patient’s and their families’ quality of life.

A recent systematic review investigated the ASD prevalence from 1993 to 2019, and found that it is currently around 0.8% in Europe, 0.95% in North America and 1.12% in Oceania, indicating a worldwide increase over the last 25 years (20). A more recent study done between 2019 and 2022 revealed a prevalence of 0,33% (1:30) among children and adolescents aged 3 to 17 years old in the United States (21). The continuous demand for a variety of treatments and the frequent lack of independence of severe ASD patients result in significant economic effects. This economic burden affects families of children and adults with ASD, the community in general, and their governments (22). The mental health of parents and caregivers is also impacted, often leading to enduring mood disorders (23).

Conventional treatments for ASD have limited benefits over symptoms related to social interaction, communication, motor function and intellectual development (23, 24). The first line of treatment consists of behavioral therapy and pharmacological interventions with anxiolytics, antidepressants, antipsychotics, mood stabilizers, alfa−2-agonists and psychostimulants (16, 25–38). Those medications may entail severe side effects, such as metabolic and endocrine dysregulation (resulting in weight gain, gynecomastia, and sexual dysfunction), plus extrapyramidal and cardiac side effects (30, 39–46). The only two medications specifically approved by FDA for ASD treatment, Risperidone and Aripiprazole, are antipsychotics indicated for management of aberrant behaviors (47). According to recent systematic reviews, both are effective for the short-term treatment of emotional dysregulation and irritability, but both are also frequently associated with important metabolic side effects (48, 49).

The endocannabinoid system plays a key role in ASD (50–71). Accordingly, recent prospective and retrospective studies of cannabinoid treatment in humans have shown promising results, with some improvements in most core ASD symptoms, as well as in several comorbid symptoms, and infrequent mild side effects (61, 72–78). The number of patients who benefit from the treatment and the level of improvement varies considerably between studies; this might be due to sample diversity and differences in the *Cannabis* extracts used, posology, outcome evaluation methodology, and treatment time.

The relative composition of cannabidiol (CBD) and Δ^9^-tetrahydrocannabinol (THC) present in the whole *Cannabis sativa* oil extracts, also known as full-spectrum *Cannabis* extracts (FCE) employed, is likely to be another relevant factor for treatment outcome. However, full-spectrum *Cannabis* extracts have complex and diverse compositions, not unlike the varied forms in which ASD conditions manifest. Henceforth, it is reasonable to speculate that most individual conditions within the autistic spectrum may benefit from a particular *Cannabis* extract composition and posology–even though some individuals may not benefit from it at all.

While some researchers believe that CBD-rich or pure CBD (CBD-CE) extracts may be the most beneficial for treating core symptoms of ASD (79–81), available data do not conclusively support this, and parameters for the clinical use of cannabinoids in ASD are yet to be established in the scientific literature. To the best of our knowledge, most studies have employed standardized Full-spectrum (whole) *Cannabis* Extracts (FCE), some with CBD to THC ratio as high as 75:1 (76), and others as low as 6:1 (72), producing some level of symptomatic improvements in both extremes. Studies using intermediary proportions, such as 20:1 (72–75) and 9:1 (78) have also promoted some amelioration of symptoms. It is important to notice, though, that the treatment with higher THC proportion (6:1) resulted in good outcomes for some cases, but led to a higher frequency of cases showing important behavioral side effects (72).

Recently it has been shown that a suitable way to overcome the lack of a well-defined posology is titrating the dosage according to the patient’s response, starting with a very low dose and adjusting it as the effects are observed (77). One study in particular has shown clinical improvements in patients using a varied range of dosages and CBD to THC proportions. In all cases these improvements were confirmed by *Cannabis*-responsive biomarkers, that moved toward normal, neurotypical values (82). Altogether, these results indicate that some patients may improve with CBD-rich, full-spectrum extracts containing very low THC levels, while others may benefit from a small increase of THC. However, it seems that CBD should be always present in an amount high enough to prevent the psychotomimetic effects of THC, whereas increase of THC, if deemed necessary, should be very slow and limited to the lower effective dose.

In the Brazilian real-life context, due to persistent lack of proper regulatory systems for production, commercialization and access to *Cannabis*-derived medicine, patients have organized themselves into dozens of civil societies. Those organizations have taken the lead in the process of securing access to *Cannabis*-based medicine for patients, especially to those who cannot afford the industrial *Cannabis*-based products currently available in Brazil. Those are either imported as a finished product, or produced in Brazil from imported concentrated extracts, which become very expensive to most of the community. Some of these nonprofit organizations (NPO) have then established their own production of full-spectrum *Cannabis* extracts with quality control and standardized composition (78).

Here we describe an open label, real-life study with 20 patients who were treated with individualized dose schemes, using one FCE or a blend of two different FCEs of standardized compositions produced by three Brazilian patient societies. Patients received FCE prescription and were treated under the clinical supervision of two authors of this article (P.M. and L.R.). For most patients, the treatment’s dosage followed the same standardized protocol adapted from the current literature and the clinicians’ previous experience, adjusting FCE’s proportions of CBD and THC to find the combination best suited to each patient.

Effects were evaluated using a retrospective outcome survey with parents of patients in the spectrum that assessed the perceived effect of FCE treatment on both core and comorbid symptoms, in a more comprehensive manner than previously reported in the literature. We have evaluated the effects of *Cannabis* treatment on 10 main different groups of symptoms, plus 9 specific subcategories for abnormal behavior symptoms and 7 specific subcategories for communication and social interaction problems. We also included five additional questions regarding positive mood improvement, side effects, changes in medication use, and general quality of life for both the patients and their families. As such, we hoped to provide a more comprehensive report of FCE’s impact over autism symptoms, accounting for different ASD conditions and extract parameters.

## 2. Methods

### 2.1. Ethics approval statement

The studies involving human participants were reviewed and approved by the Ethics Committee on Human Research of the Health Sciences College of the University of Brasília (Universidade de Brasília–UnB), under the protocol number CAAE 54241721.5.0000.0030. Written informed consent to participate in this study was provided by every participant and its legal guardian/next of kin. Written informed consent for publishing of data obtained in this study was provided by the participants’ legal guardian/next of kin.

### 2.2. Participants

Treatment with FCE was conducted by two of the authors (PM and LS) in the clinical setting. Either during or after the end of each patient’s treatment, parents, or participants (according to the better convenience in each case) were invited to take part in this study. Twenty participants diagnosed with ASD agreed to participate and were enrolled. We are interested in evaluating the benefits of FCE treatment for any condition within the autistic spectrum, therefore the inclusion criterion was ASD diagnosis (ICD 10 = F84.0) previously received by the patients, or given after clinical evaluation by the authors PM or LS. ASD patients treated with FCE for at least 3 months were included. Participation consisted of allowing the disclosure of patient’s clinical records and answering a detailed survey (Supplementary material S1).

In Table 1 we indicate participant’s weights, genders and details of cannabinoid concentrations consumed daily and per kilo. Patients’ gender is identified as male (m) or female (f) numbers throughout the text and figures. In total, 118 families were initially invited to take part in the research, and 24 of those families answered the survey. Out of those, one participant was removed because he had been under less than 3 months of FCE treatment, one participant was removed due to faulty survey answers, and two others were removed because they used CEs that deviated from the scope of this paper. Of note, three of the enrolled patients chose to interrupt the treatment of their own accord: one (2 m) due to worsening of symptoms and two due to financial limitations (7 m, 19 m), but all treatments lasted 3 months or more, and their data was maintained in the analysis. Patient 16 m used a non-full-spectrum, pure CBD product. This was the only case treated throughout with a purified CBD formulation, which was eventually supplemented with THC-rich FCE.

**Table 1:** Cohort description, weight, and FCE dosage for each patient at the beginning and at the end of treatment.

Initial									Final					
Patient#	Age years	FCE treatment months	Weight kg	CBD mg/kg/day	THC mg/kg/day	CBD mg/day	THC mg/day	CBD:THC ratio	Weight kg	CBD mg/kg/day	THC mg/kg/day	CBD mg/day	THC mg/day	CBD:THC ratio
1 m^a^	05	13	20	2.25	0.10	45.00	2.07	21:1	22	3.41	0.16	75.00	3.45	21:1
2 m^a^	09	03	26	0.86	0.02	22.44	0.46	49:1	27	1.45	0.03	39.27	0.80	49:1
3 m^a^	12	13	38	1.58	0.39	60.00	15.00	4:1	40	1.88	0.47	75.00	18.75	4:1
4 m^a^	38	03	81	0.93	0.04	75.00	3.45	21:1	81	0.93	0.04	75.00	3.45	21:1
5 m^a^	14	06	50	0.90	0.23	45.00	11.25	4:1	55	1.09	0.27	60.00	15.00	4:1
6m^b^*	16	09	55	1.36	0.06	75.00	3.45	21:1	55	1.37	0.10	75.09	5.45	14:01
7 m^a^	20	19	56	0.32	0.04	18.00	2.40	7.5:1	62	0.29	0.04	18.00	2.40	7.5:1
8m^c^*	14	06	64	0.59	0.06	37.59	3.80	11:1	65	0.58	0.05	37.57	3.30	11:1
9 m^a^	05	11	14	3.21	0.80	45.00	11.25	4:1	15	5.00	1.25	75.00	18.75	4:1
10m^b^*	22	20	44	0.77	0.02	33.66	0.68	5:1	50	0.76	0.16	37.77	7.80	5:1
11 m^a^	06	09	22	3.41	0.85	75.00	18.75	4:1	22	5.45	1.36	120.00	30.00	4:1
12 m^a^	09	11	26	2.88	0.72	75.00	18.75	4:1	23.6	3.81	0.95	90.00	22.50	4:1
13 m^a^	11	04	65	1.15	0.29	75.00	18.75	4:1	63	0.71	0.18	45.00	11.25	4:1
14 m^a^	10	16	43	0.65	0.01	28.05	0.57	4:1	43	2.09	0.52	90.00	22.50	4:1
15f^c^*	19	18	32	0.35	0.10	11.36	3.23	3:1	40	2.25	0.64	90.14	25.50	3:1
16m^b^*^&^	22	12	70	4.29	0.00	300.00	0.00	20:1	72	3.34	0.17	240.54	12.00	20:1
17f^a^	08	20	14	0.81	0.03	11.28	0.48	21:1	18	2.08	0.10	37.50	1.80	21:1
18 m^a^	25	07	79	0.95	0.24	75.00	18.75	4:1	68	2.65	0.66	180.00	45.00	4:1
19 m^a^	19	04	90	0.50	0.01	44.88	0.91	49:1	75	1.50	0.03	112.20	2.28	49:1
20 m^a^	04	21	12.2	2.76	0.06	33.66	0.68	49:1	12.4	3.62	0.07	44.88	0.91	49:1
														
Average	14.4	11.25	43.50	0.93	0.06	46.63	7.09	7:1	46.50	1.88	0.16	72.50	12.68	6:1
STD	8.44	6.10	24.29	1.17	0.28	23.19	7.42	3:1	22.09	1.47	0.41	52.60	11.99	4:1

Patients were identified as male (m) or female (f) next to their randomly assigned numbers. According to initial symptoms and responses to the treatment, patients have received one out of three general formulation schemes as follows: fifteen patients have used only CBD-rich FCEs throughout treatment (A); three patients started with a CBD-rich FCE, which was eventually supplemented with THC-rich FCE (B); and two patients have used a blend of a CBD-rich FCE with a THC-rich FCE throughout treatment (C). *Patients that have used a THC-enriched FCE at some point during the treatment. One of the formulations (&) did not include THC, therefore it was not included in the statistics for CBD to THC proportion.

### 2.3. Treatment

Most patients received individualized treatments based on a titration protocol, which started with low doses of a CBD-rich FCE. According to the clinical evaluation of observed effects, the dose was slowly increased and could be gradually supplemented by low doses of a THC-rich FCE to improve the results. We define “CBD-rich” FCEs as those in which CBD amount is higher than THC, and “THC-rich” FCEs as those in which THC is the more abundant cannabinoid. The CBD-rich FCEs had a proportion of either 4: 1, 21: 1, 49: 1 or 7: 1 (CBD: THC), whereas THC-rich FCEs had a proportion of either 1: 22 or 1: 4 (CBD: THC, rounded to whole numbers). One exceptional case started treatment with a purified, industrial grade CBD extract (identified in Table 1), but eventually added a FCE rich in THC. Two patients presenting refractory severe behavioral symptoms have used a blend of CBD-rich and THC-rich extracts since the beginning of the treatment. These extracts were produced by three patient organizations, and the specific extracts used by each patient depended on their choice of affiliation to a specific NPO. All extracts were administered orally via drops. Thus, daily doses were varied, changed according to symptoms and side effects presented by the patient during treatment, and eventually stabilized after reaching a consensus between physician and family.

**Figure 1:**
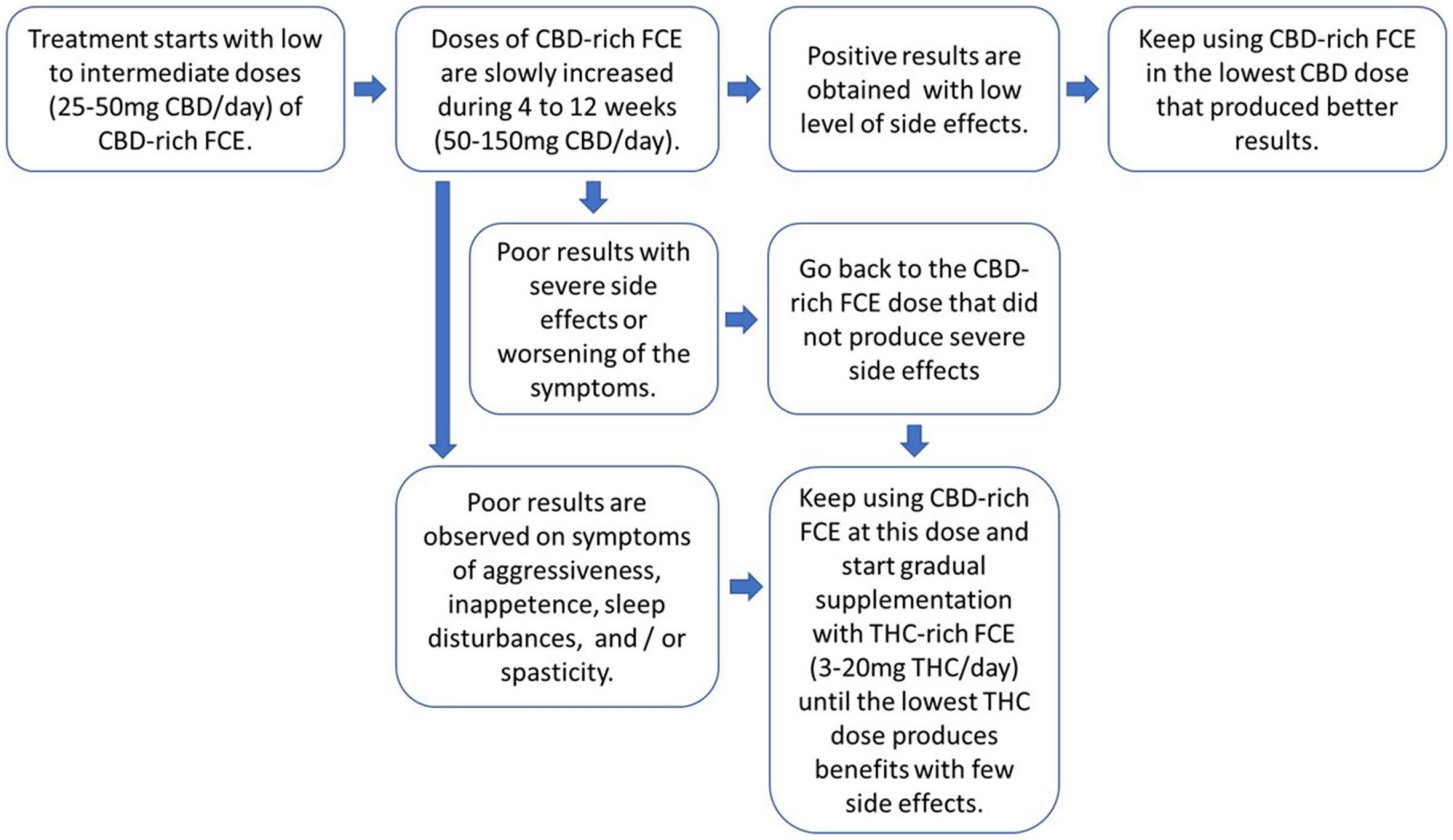
General guidelines for individual tailoring of FCE posology and CBD to THC proportions. The protocol should aways start with low doses of CBD-rich FCEs for all patients and doses should be slowly increased until improvements are observed. Doses can be divided in two or three daily administrations. If improvements are not observed and/or side-effects begin to occur, return to the dosage of the CBD-rich FCE that showed no side effects and start supplementing it with a THC-rich FCE, gradually increasing the final the proportion of THC resulting from the blend of the two FCEs.

Patients were evaluated monthly for a trimester. Upon symptoms stabilization, they were reevaluated every 6 months. In rare cases weekly reassessments/adjustments were made. Criteria employed for CBD-rich FCE dosage change, or supplementation with THC-rich FCE were: (1) severe cases of psychomotor agitation and aggression with little response to CBD; (2) persistence of complaints regarding sleep pattern and appetite despite otherwise satisfactory response to CBD (More details in Figure 1). Average concentrations at the beginning of treatment were 45 mg/day: 1.94 mg/day (CBD: THC) and 75 mg/day: 2.32 mg/day at the end of treatment, normally administered in two or three daily doses.

### 2.4. Cannabinoid extracts

*Cannabis* extracts were produced and distributed to all the patients in the study by three Brazilian patient NPOs, the Brazilian Society of Medicinal Cannabis Patients (also known as AMAME) the Cannabic Society in Defense of Life (also known as Maria Flor) and the Brazilian Support Society *Cannabis Hope* (also known as ABRACE). The quality and composition of cannabis extract samples were assessed by high-performance liquid chromatography (HPLC) analysis performed at certified laboratories.

### 2.5. Parents-reported outcome survey

The treatment reported here resulted from an adaptation of available clinical data to the Brazilian reality, but it was not originally intended to produce scientific data. Therefore, the approach used by each clinician contributing to this report to assess baseline clinical indicators of the ASD patients was not the same, which precluded us from using a before-*vs*-after approach. Thus, we have used a structured, caregiver-reported single-assessment retrospective outcome survey sent by e-mail to the patients and their families (Supplementary material). The questionnaire was adapted from the methodology previously used by Fleury and colleagues (76). Thus, we have employed a Likert-like scale (83) composed of six options to evaluate the perceived outcome of the treatment for 10 main symptom categories, 16 specific sub-categories for abnormal behavior and communication/social interaction impairments, and five additional non-symptomatic aspects. Parents were explicitly asked to report the difference observed since FCE treatment onset. The options for each question were: “Does Not Apply,” “Considerable Worsening,” “Moderate Worsening,” “No Change,” “Moderate Improvement” and “Considerable Improvement.”

In addition, responders were asked to describe in their own words the changes observed for each symptom category to ensure that the responder properly understood the meaning of each category and that they were scoring the Likert-like scale in a consistent way throughout the study. If discrepancies were detected, the responders were contacted by telephone or text messages to further discuss the definition of that specific symptom, and then they were asked whether they wished to reconsider their answers. These open-ended questions also helped to elucidate more practical and subjective impacts of the treatment (see discussion). The parents were invited to answer the survey from March 2022 to August 2022. Clinical charts of each patient were also assessed to obtain details of the treatment, such as weight, doses and use of other medications.

The 10 main symptom categories and five non-symptomatic aspects evaluated in the survey were chosen after reviewing their prevalence in the literature, adapting some of the available instruments (mainly the Autism Treatment Evaluation Checklist, ATEC), and considering the clinical experience of the physicians and ASD patient’s parents. Symptom categories used are as follows: (1) Attention deficit and hyperactivity disorder; (2) Abnormal behaviors; (3) Sadness, melancholy, bad moods; (4) Impaired motor development and motor coordination; (5) Lack of independence for daily activities; (6) Impaired communication and personal interactions (verbal and non-verbal); (7) Intellectual and cognitive deficits; (8) Sleep issues; (9) Seizures; (10) Avoidance and/or restrictions to food intake; (11) Positive mood states; (12) Overall patient’s quality of life; (13) Adverse effects due to treatment; (14) Use of other medications; (15) Overall family’s quality of life.

In the main group of categories, concerning Abnormal behavior, and Impaired communication and verbal interactions, each survey responder is likely to evaluate the outcome in each of these two categories considering their own reality regarding the intensity and scope of behavioral and communication problems of the patient. Further outcome details regarding these aspects were collected in two separated groups. Thus, the category “abnormal behaviors” was further subdivided into nine specific aspects as follows: (1) Stereotypies; (2) Aggressiveness Toward Others; (3) Self-Aggressiveness; (4) Autistic Meltdown Crisis/Temper Tantrum; (5) Screams and Random Sounds; (6) Obsessive Compulsive Behaviors; (7) Eating Non-Foods; (8) Discomfort in Noisy/Crowded Places; (9) Excessive Appetite.

In Communication and Social Interactions, we also analyzed specific related features, as follows: (1) Impaired Verbal Communication; (2) Impaired Visual Contact; (3) Impaired Response to Their Own Name; (4) Impaired Attention to Receptive Direct Verbal Communication; (5) Production of Sounds or Isolated Words with Communicative Function; (6) Impaired Written Communication; (7) Use of Alternative Forms of Communication (gestures, signals, cards, software applications, and other systems with images).

Adverse effects and Use of other medications were not evaluated using the above-mentioned scale, but by questions with open written answers, multiple-choice questions and by consulting patients’ medical records.

### 2.6. Statistical analysis

**Figure 2:**
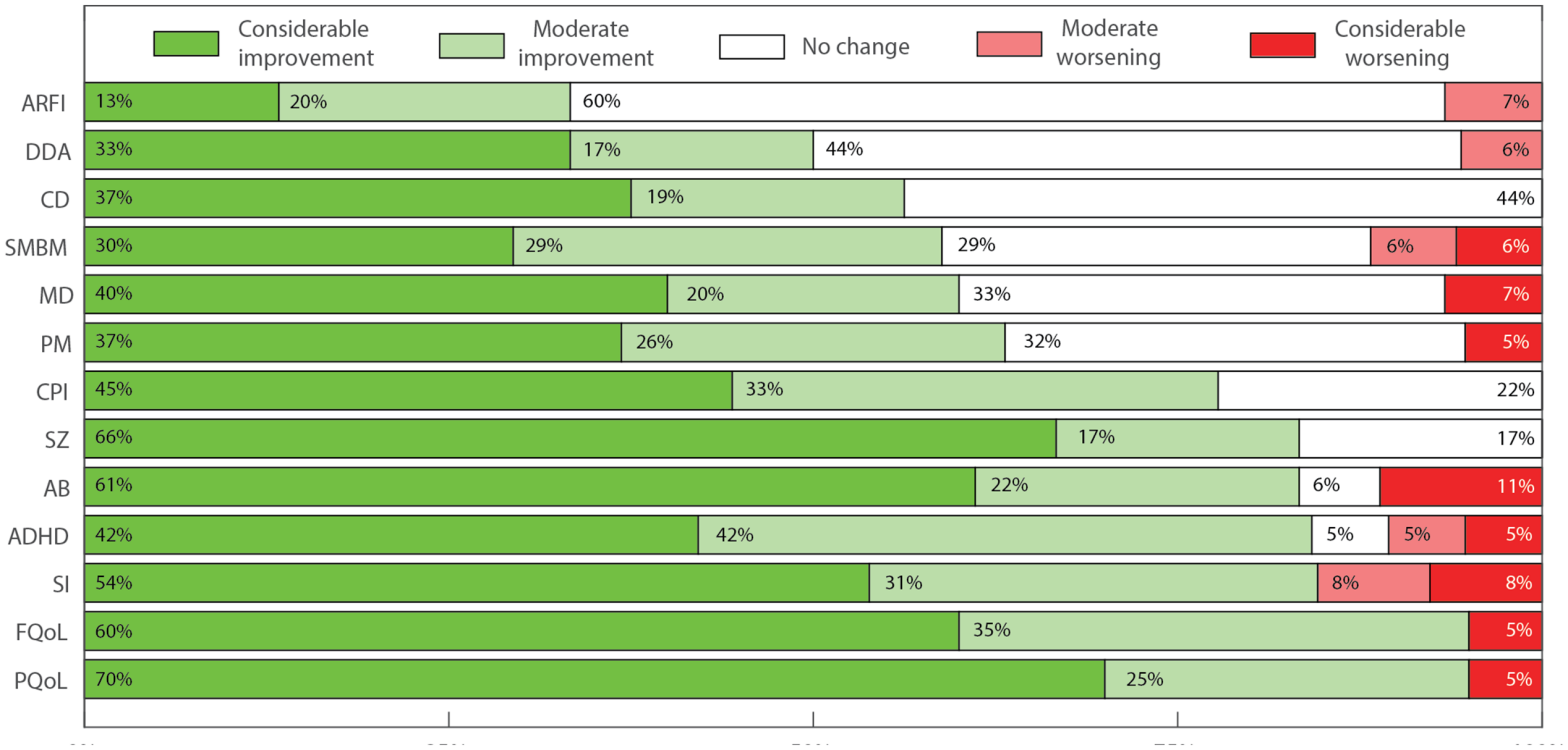
Perceived effects of FCE treatment over main symptom categories and aspects of ASD as percentages of the cohort. Percentages were rounded to whole numbers for clarity. Avoidance and/or restrictions of food intake (ARFI, *n* = 15); Lack of independence for daily activities (DDA, *n* = 18); Intellectual and cognitive performance deficits (CD, *n* = 16); Sadness, melancholy and bad moods (SMBM, *n* = 17); Impaired motor development and motor coordination (MD, *n* = 15); Positive mood states (PM, *n* = 19); Impaired communication and personal interactions (verbal and non-verbal; CPI, *n* = 18); Seizures (SZ, *n* = 6); Abnormal behaviors in general (AB, *n* = 18); Attention deficits/ hyperactivity disorder (ADHD, *n* = 19); Sleep issues (SI, *n* = 13); Overall family’s quality of life (FQoL, *n* = 20); Overall patient’s quality of life (PQoL, *n* = 20).

**Figure 3:**
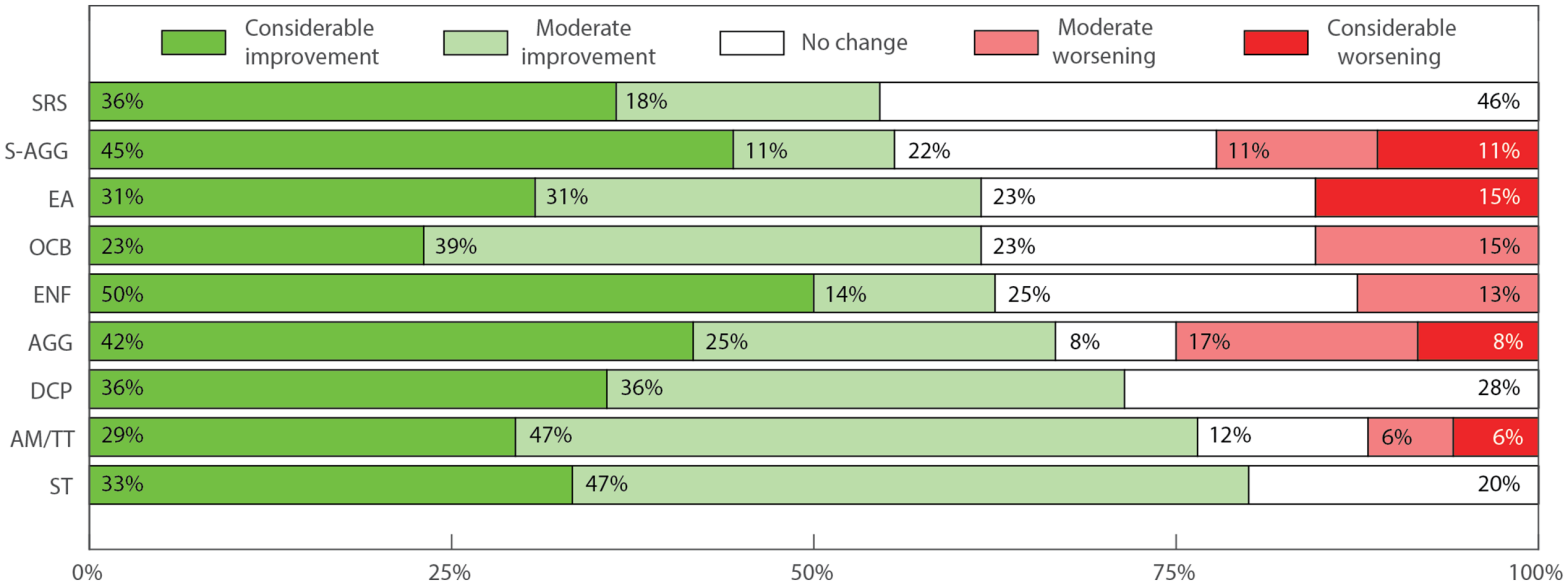
Perceived change in sub aspects of abnormal behaviors. Percentages were rounded to whole numbers for clarity. Screams and Random Sounds (SRS, *n* = 11); Self-Aggressiveness (S-AGG, *n* = 9); Excessive Appetite (EA, *n* = 13); Obsessive compulsive behaviors (OCB, *n* = 13); Eating non-foods (ENF, *n* = 8); Aggressiveness toward others (AGG, *n* = 12); Discomfort in noisy/crowded places (DCP, *n* = 14); Autistic Meltdown crisis/temper tantrum (AM/TT, *n* = 17); Stereotypies (ST, *n* = 20). Percentages were rounded to whole numbers for clarity.

**Figure 4:**
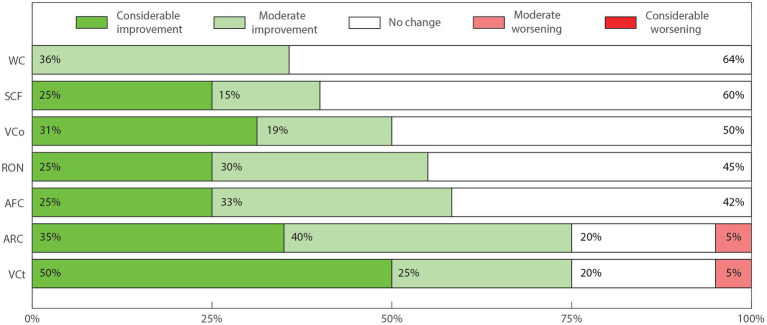
Perceived change in sub-categories of communication and interaction impairments. Impaired written communication (WC, *n* = 14); Production of sounds or isolated words with communicative function (SCF, *n* = 20); Impaired verbal communication (VCo, *n* = 16); Impaired response to their own name (RON, *n* = 20); Use of alternative forms of communication (AFC, *n* = 12); Impaired attention to receptive direct verbal communication (ARC, *n* = 20); Impaired visual contact (VCt, 20).

To generate descriptive outcome scores suitable for interpatient comparisons and statistical analysis, numeric labels were assigned to each of the five possible outcome answers seen in the Parents Outcome Survey. Namely, −2; −1; 0; 1; 2 for the scale of effects, and the # label was assigned when the patient did not present the specific symptom evaluated. The total number of patients for each symptom or aspect is equal to our cohort number minus the number of people who answered “Did Not Apply” (20 – #). Numbers in Figures 2–4 are shown as percentages of the whole cohort that presented each symptom or non-symptomatic aspect, plotted using MATLAB R2022a (Figures 2–4). Three outcome scores were generated, namely: General Outcome Score (GOS), an average of the scores from categories 1 to 11; Abnormal Behaviors Outcome Score (ABOS), an average of the scores from abnormal behavior sub-categories; and Communication and Interaction Outcome Score (CIOS), an average of the scores from communication and interaction sub-categories. Each score includes only the symptoms or categories presented by each patient, and each symptom or category present has the same weight.

## 3. Results

### 3.1. General results

Concerning the dosage/formulation scheme, among the patients who answered the survey, 15 patients have used only CBD-rich FCEs throughout treatment (a); three patients started with a CBD-rich FCE, which was eventually supplemented with THC-rich FCE (b); and two patients have used a blend of a CBD-rich FCE with a THC-rich FCE throughout treatment (c). This may reflect a similar distribution pattern present in the whole treated population, but it actually emerged as a direct result of who chose to answer the survey or not. Among the predominant group who exclusively used CBD-rich FCEs throughout the treatment, only one patient, 2 m, showed a negative GOS (−1.1), whereas the other 14 showed positive GOS. All three patients who started with only CBD-rich FCE and eventually supplemented it with a THC-rich FCE, showed positive GOS. One of the two patients who used a blend of CBD-rich and THC-rich FCEs since the beginning, 8 m, showed positive GOS (1.0), while the other, 15f, showed a slightly negative GOS (−0,3). Overall, only 2 patients did not show positive GOS. This is a very high proportion of improvement, even though we cannot know whether this reflects the reality of the whole treated population, or the result of some bias, which, for instance, could have motivated parents who perceived better results to be more willing to answer the very detailed survey used here. Out of the 20 patients included in the study, 16 (80%) were treated with FCE for 6 months or more, and the average FCE treatment duration was 11.25 (± 6.1 SD) months. Average age was 14 years old (5–38) at the moment of the survey, being 18 males and 2 females. Average doses CBD changed from 46.63 (± 23.19 SD) mg/day at the beginning to 72.50 (± 37.82 SD) mg/day at the end, while the average dose of THC changed from 7.09 (± 7.42 SD) mg/day to 12.68 (± 12.31 SD) mg/day, representing an increase of average dose/day of 25.87 mg and 5.59 mg, respectively. The average final doses per weight were 1.88 (± 1.47 SD) mg/kg/day of CBD and 0.16 (± 0.41 SD) mg/kg/day of THC. The average final CBD to THC proportion was 5.71 (± 3.07 SD):1.

In Tables 2 3 and 4 we show the raw data from the survey answers of all participants regarding perceived effects on the main symptoms and on the symptoms subcategories. In cases when a given symptom/aspect did not apply to that patient, response was marked with a hashtag (#). Adverse Effects and Use of Other Medication are shown in Table 5 due to their particular type of answer (see Supplementary material).

**Table 2:** Perceived improvement of symptoms and non-symptomatic aspects after FCE treatment for each patient.

Case #	FCE treatment Months	Perception of improvement (main symptom categories and quality of life)															ADHD	AB	SMBM	MD	LIDA	CPI	CD	SI	SZ	ARFI	PM	GOS	PQoL	FQoL
1 m	13	1	1	1	1	1	2	1	2	#	1	1	1.2	2	1
2 m	3	-2	-2	-2	0	0	#	#	-1	#	0	-2	−1.1	−2	−2
3 m	13	0	#	0	#	#	#	#	1	#	0	#	0.3	1	1
4 m	3	1	2	1	#	0	1	#	1	#	#	1	1.0	1	1
5 m	6	2	2	0	0	#	1	0	2	#	1	1	1.0	1	1
6 m*	9	1	2	0	0	0	0	0	1	#	0	1	0.5	2	2
7 m	19	2	2	1	0	2	0	2	2	#	0	2	1.3	2	2
8 m*	6	2	2	#	#	0	1	0	#	#	2	0	1.0	1	1
9 m	11	#	2	2	#	1	1	#	#	#	0	2	1.3	2	2
10 m*	20	1	2	2	2	2	2	0	1	#	2	0	1.4	2	2
11 m	9	1	2	#	2	2	2	2	#	#	#	0	1.6	2	2
12 m	11	2	2	2	2	2	2	2	#	#	1	2	1.9	2	2
13 m	4	1	1	0	#	−1	2	1	#	#	#	0	0.6	1	1
14 m	16	2	1	1	2	0	1	0	#	#	0	2	1.0	2	2
15f*	18	−1	−2	−1	−2	0	2	1	−2	1	0	1	−0.3	2	1
16 m*	12	1	1	1	2	2	1	2	2	2	#	2	1.6	2	2
17f	20	1	2	2	1	2	2	0	2	2	−1	2	1.4	2	2
18 m	7	2	#	#	2	1	2	2	#	2	#	0	1.6	2	2
19 m	4	2	2	2	1	0	0	2	2	0	0	2	1.2	2	2
20 m	21	2	0	0	0	0	0	0	2	2	0	0	0.5	2	2
n	20	19	18	17	15	18	18	16	13	6	15	19	20	20	20
Median	11.0	1	2	1	1	0.5	1	1	2	2	0	1	1.1	2	2
Mean	11.3	1.1	1.2	0.7	0.9	0.8	1.2	0.9	1.2	1.5	0.4	0.9	0.9	1.6	1.5

Attention deficits/hyperactivity disorder (ADHD, *n* = 19); abnormal behaviors (AB, *n* = 18); sadness, melancholy and bad mood (SMBM, *n* = 17); impaired motor development and motor coordination (MD, *n* = 15); lack of independence for daily activities (LIDA, *n* = 18); impaired communication and personal interactions (verbal and non-verbal) (CPI, *n* = 18); intellectual and cognitive performance deficits (CD, *n* = 16); sleep issues (SI, *n* = 13); seizures (SZ, *n* = 6); avoidance and/or restrictions of food intake (ARFI, *n* = 15); positive mood states (PM, *n* = 19); overall patient’s quality of life (PQoL, *n* = 20); overall family’s quality of life (FQoL, *n* = 20). *Patients that have used a THC-enriched FCE at some point during the treatment. Outcome scale used: # “does not apply,” −2 “considerable worsening,” −1 “moderate worsening,” 0 “no change,” 1 “moderate improvement” and 2 “considerable improvement.” GOS, general outcome score.

**Table 3:** Perceived improvement of abnormal behavior sub-aspects after FCE treatment for each patient.

Case #	FCE treatment months	Perception of improvement of abnormal behavioral symptoms sub-categories										ST	AGG	S-AGG	AM/TT	SRS	OCB	ENF	DCP	EA	ABOS
1 m	13	2	2	#	2	2	1	#	2	1	1.8
2 m	3	0	−2	#	−2	#	0	#	0	#	−0.7
3 m	13	#	#	#	0	#	0	#	#	#	0.0
4 m	3	1	1	0	1	#	2	#	1	0	0.9
5 m	6	#	#	#	1	#	#	2	1	#	1.3
6 m*	9	1	1	#	2	0	−1	2	0	1	0.7
7 m	19	1	1	1	0	#	#	#	0	0	0.4
8 m*	6	1	2	#	1	1	2	2	#	1	1.4
9 m	11	#	#	#	1	2	#	0	#	#	0.8
10 m*	20	0	#	2	1	0	#	#	2	2	1.3
11 m	9	1	#	#	1	#	#	#	#	−2	0.0
12 m	11	2	2	2	2	1	1	1	1	#	1.6
13 m	4	1	−1	−1	1	2	1	0	#	0	0.4
14 m	16	2	−1	0	1	0	1	−1	2	2	0.8
15f*	18	0	0	−2	−1	0	−1	#	#	1	−0.3
16 m*	12	2	#	#	#	#	#	#	1	−2	0.3
17f	20	#	2	2	2	#	0	#	2	#	1.7
18 m	7	1	#	#	#	0	1	#	2	2	1.2
19 m	4	2	2	2	2	2	2	2	1	2	1.9
20 m	21	#	#	#	#	#	#	#	0	#	0.0
n	20	15	12	9	17	11	13	8	14	13	20
median	11	1	1	1	1	1	1	1	1	1	0.8
mean	11.3	1.0	0.0	0.7	0.9	0.9	0.7	1.0	1.0	0.6	0.8

ST, Stereotypies; AGG, aggressiveness toward others; S-AGG, self-aggressiveness; AM/TT, autistic meltdown crisis/temper tantrum; SRS, screams and random sounds; OCB, obsessive compulsive behaviors; ENF, eating non-foods; DCP, discomfort in noisy/crowded places; EA, excessive appetite. ABOS, abnormal behavior outcome score. Outcome scale used: # does not apply, −2 considerable worsening, −1 moderate worsening, 0 no change, 1 moderate improvement and 2 considerable improvement. GOS, general outcome score. *Patients that have used a THC-enriched FCE at some point during the treatment.

**Table 4:** Perception of improvement in sub-aspects on impaired communication and interaction after FCE treatment for each patient.

Case #	FCE treatment months	Perception of improvement in sub-aspects on impaired communication and interaction								VCo	VCt	RON	ARC	SCF	WC	AFC	CIOS
1 m	13	2	2	2	2	1	1	2	1.7
2 m	3	#	0	0	−1	0	#	#	−0.3
3 m	13	#	0	0	0	0	#	#	0.0
4 m	3	1	1	0	0	0	1	0	0.4
5 m	6	1	1	0	1	1	0	#	0.7
6 m*	9	0	2	1	1	0	0	0	0.6
7 m	19	#	0	0	0	0	0	#	0.0
8 m*	6	1	1	1	1	1	0	0	0.7
9 m	11	0	2	0	1	0	#	#	0.6
10 m*	20	0	2	1	2	2	0	2	1.3
11 m	9	2	1	1	1	2	1	1	1.3
12 m	11	2	2	2	2	2	1	2	1.9
13 m	4	2	0	0	0	2	1	1	0.9
14 m	16	0	2	2	2	0	0	0	0.9
15f*	18	0	2	1	1	0	#	#	0.8
16 m*	12	#	1	1	1	0	#	#	0.8
17f	20	0	2	2	2	0	#	1	1.2
18 m	7	2	2	2	2	2	0	1	1.6
19 m	4	0	2	0	2	0	0	#	0.7
20 m	21	0	−1	0	1	0	0	0	0.0
n	20	16	20	20	20	20	14	12	20.0
Median	11	1	2	1	1	0	0	1	0.7
Mean	11.2	0.8	1.2	0.8	1.1	0.7	0.4	0.8	0.8

VCo, Impaired verbal communication; VCt, impaired visual contact; RON, impaired response to their own name; ARC, impaired attention to receptive direct verbal communication; SCF, production of sounds or isolated words with communicative function; WC, impaired written communication; AFC, use of alternative forms of communication. CIOS, communication and interaction outcome score. *Patients that have used a THC-enriched FCE at some point during the treatment. Outcome scale used: # does not apply, −2 considerable worsening, −1 moderate worsening, 0 no change, 1 moderate improvement and 2 considerable improvement. GOS, general outcome score.

**Table 5:** Medications other than FCE used by the patients, and untoward effects observed during treatment with FCE.

Case *#*	GOS	ABOS	CIOS	Medication before FCE treatment	Medication after FCE treatment	Summary of changes	FCE side effects
1 m	1.2	1.8	1.7	N	N	N	Agitation, Drooping eyelids
2 m	−1.1	−0.7	−0.3	Risperidone, Buspirone, Methylphenidate	N	CWD	Agitation, Difficulty sleeping
3 m	0.3	0.0	0.0	Sertraline, Aripiprazole	Sertraline, Aripiprazole	ID for both	Agitation, Difficulty sleeping
4 m	1.0	0.9	0.4	N	N	N	Cough
5 m	1.0	1.3	0.7	Methylphenidate	Methylphenidate	DR	N
6 m*	0.5	0.7	0.6	Risperidone	Risperidone	DR	Eye redness, Excessive thirst
7 m	1.3	0.4	0.0	Sertraline, Risperidone	Sertraline, Risperidone	DR for Risperidone	Agitation, excessive thirst
8 m*	1.0	1.4	0.7	Risperidone	Risperidone	N	N
9 m	1.3	0.8	0.6	N	N	N	N
10 m*	1.4	1.3	1.3	Aripiprazole, Valproate, Naltrexone, Clorpromazine, Biperiden	N	CWD	Urinary incontinence, Excessive appetite, Weight gain
11 m	1.6	0.0	1.3	N	N	N	Agitation, Excessive appetite
12 m	1.9	1.6	1.9	Risperidone, Methylphenidate, Belladona	N	CWD	Reduced appetite, Weight loss, vomiting
13 m	0.6	0.4	0.9	Aripiprazole, Chlonidine	Aripiprazole, Chlonidine	DR for both	Agitation
14 m	1.0	0.8	0.9	Respiridon	N	CWD	N
15f*	−0.3	−0.3	0.8	Oxcarbazepin, Clobazam, Topiramate, Periciazine	Oxcarbazepin, Clobazam, Topiramate, Periciazine	ID for all	Difficulty sleeping, Weight gain
16 m*	1.6	0.3	0.8	Valproate, Aripiprazole, Fluouxetin	Fluoxetin	WD of Valproate, Aripiprazole	Agitation, Excessive thirst
17f	1.4	1.7	1.2	Clobazam, Valproate, Levetiracetam, Lamotrigine	N	CWD	N
18 m	1.6	1.2	1.6	Valproate, Lacosamide, Levetiracetam	Valproate, Lacosamide	WD of Levetiracetam and DR of the others	Eye redness, Excessive thirst, weight loss
19 m	1.2	1.9	0.7	Quetiapine, Valproate, Lamotrigine, Bupropione, Risperidone	Valproate, Bupropione	WD of Quetiapine, Lamotrigine, Risperidone	N
20 m	0.5	0.0	0.0	Valproate, Lamotrigine	N	CWD	Excessive sleep, Constipation, Excessive thirst, Reduced appetite, Weight loss

GOS, General Outcome Score; ABOS, abnormal behavior outcome score. CIOS, communication and interaction outcome score; N, none; WD, withdrawal; CWD, complete withdrawal; DR, dosage reduction; ID, increased dosage; *Patients that have used a THC-enriched FCE at some point during the treatment.

### 3.2. Results grouped by symptom category

There was a general perception of improvement for all 10 main symptom categories and 3 non-symptomatic aspects analyzed (Figure 2). Some features improved more frequently than others. While “avoidance and/or restrictions to food intake” improved in approximately 30% of cases, ADHD symptoms, “behavioral disorders,” “communication and social interaction deficits,” “sleep disorders,” “seizures,” “patient’s quality of life” and “family’s quality of life” improved in at least 77% of cases. General improvement in “intellectual and cognitive performance” were observed in 57% of the cases, which is very striking since there is no other pharmacological treatment known to improve this impactful and recurrent aspect of severe ASD.

Benefits from using FCE were observed in 84% of seizure cases, in general accordance with many other reports from the literature (84). Considerable improvement was equal or more frequent than 33% in 12 out of the 13 main categories (Table 2 and Figure 2). Remarkably, “patient’s quality of life” and “family’s quality of life” improved in 95 and 83% of cases, respectively. Only two patients, 2 m and 15f presented an overall worsening or no change in the main symptom categories (GOS equal to −0,3 and − 1.1, respectively).

### 3.3. Sub-categories of abnormal behaviors

There was improvement in at least 54% of cases in all 9 sub-categories of abnormal behaviors, as indicated in Table 3. A graphic representation of these results is presented in Figure 3. The frequency of patients presenting overall improvement in each of the specific subcategories of abnormal behavior was as follows: “stereotypies” 80%; “autistic meltdown crisis/temper tantrum” 76%; “discomfort in noisy/crowded places” 72%; “aggressiveness toward others” 67%; “eating non-foods” 63%; “excessive appetite” 62%; “obsessive compulsive behaviors” 62%; “self-aggressiveness” 56%; and “screams and random sounds” 54%. The frequency of “considerable improvement” perception was above 30% for 8 out of the 9 sub-categories, and ranged from 23% for “obsessive compulsive behavior” to 50% for “eating non-foods.”

Worsening of one or more abnormal behavior sub-categories was perceived in 7 out of the 20 patients (35%), with 3 out of 12 (25.0%) reporting worsening in “aggressiveness toward others”; 2 out of 9 (22.2%) in “self-aggressiveness,” 2 out of 17 (11.8%) in “meltdown crisis/temper tantrum,” 2 out of 13 (15.4%) in “obsessive compulsive behaviors,” 1 out of 8 (12.5%) in “eating non-foods” and 2 out of 13 (15.4%) in “excessive appetite.” “Considerable worsening” was perceived in 5 out of 20 patients (25.0%) in four sub-categories, with 1 out of 12 (83.3%) reporting considerable worsening in “aggressiveness toward others,” 1 out of 9 (11.1%) in “self-aggressiveness,” 1 out of 17 (5.8%) in “meltdown crises/temper tantrums” and 2 out of 13 (15.3%) in “excessive eating.” Nevertheless, only two patients, 2 m and 15f presented an overall worsening in abnormal behavior sub-categories (ABOS equal to −0.3 and − 0.7, respectively).

### 3.4. Sub-categories of communication and social interactions

There was consistent improvement in all communication and social interaction sub-categories. We highlight that “attention to receptive direct verbal communication” improved in 17 out of 20 (85%), while “visual contact” and “attention to conversation” improved in 15 out of 20 (75%) of cases. “Verbal communication” improved in 8 out of 16 (50%) of cases, “response to their own name” improved in 11 out of 20 (55%) of cases, “written communication” improved in 5 out of 14 (35.7%) of cases and “alternative forms of communication” improved in 7 out of 12 (58%) of cases. Actual worsening of cases was found only in one case for “visual contact” and in one case for “attention to receptive direct verbal communication.” Only patient 2 m presented an overall worsening in communication and interaction sub-categories (CIOS equal to −0.3).

### 3.5. Other medications used during FCE treatment

Medications other than the FCE used by the patients are indicated in Table 5. As FCE treatment progressed, out of the 16 patients receiving other neuropsychiatric medications, four (25%) reduced the dosage for at least one type of medication, nine (56%) discontinued at least one type of medication, and six (37%) discontinued all medication. Therefore, out of 16 patients, 13 (81%) have reduced or discontinued neuropsychiatric medications after using FCE. Only two patients increased the doses of medications other than FCE during treatment, and one patient’s medications remained unaltered.

### 3.6. Untoward effects observed during CE treatment

The adverse effects reported in our cohort of patients are also shown in Table 5. In most cases, adverse effects were mild and temporary. The most frequent adverse effects were agitation (6), difficulty sleeping (3), excessive thirst (3), eye redness (2), excessive appetite (2), weight gain (2), reduced appetite (2), weight loss (2). Only one patient (2 m) abandoned the treatment due to worsening of symptoms.

## 4. Discussion

### 4.1. General results

In this manuscript we present the perceived improvements of ASD patients and their families over the course of 3–21 months of treatment with FCE, using a titration protocol that resulted in personalized CBD and THC dosages, resulting in individually tailored CBD to THC proportions ranging from 49:1 to 1:22. Our sample consisted of 20 patients of varied ages and ASD severities. Parents reported improvement in all aspects evaluated. Side effects observed during FCE treatment were mild, as well as untoward interactions with other medications. As treatment evolved, most of other medications had their doses reduced or were completely removed during FCE treatment, which is consistent with the subjective perception of general improvement after FCE treatment. Patients’ and their families’ quality of life improved in 19 out of the 20 cases.

Difficulties in language and social interaction from a young age can result in behavioral issues, poor social skills and the consequent impacts over the quality of life of ASD patients (85). An overall perception of improvement in the main category “impaired communication and personal interactions” (CPI) was perceived in 78% of the patients (Figure 2). Accordingly, our analysis of the sub-categories of communication and interaction yielded a positive average communication and interaction outcome score (CIOS) of 0.8 (Table 4). The positive results were mostly driven specifically by benefits in speech, visual contact, and attention (Figure 4). In their open answers to our control questions (see Supplementary material), parents often reported feeling happy to see their children actively engaging with them, as well as with colleagues in the school setting during FCE treatment. This reinforces the increasing amount of data supporting the use of FCE to treat some of the most impactful core aspects of ASD (8), which are not effectively treated by any of the currently available medications.

Here we also provide a more detailed account on the effect of FCE treatment for the well-being of people with autism and the nuances of non-core symptoms. The generally positive outcome in social interaction, cognition and behavioral abnormalities seems to have had very impacting effects over quality of life of both families and patients, which are of particular interest, reflecting the benefits of FCE in a more comprehensive way. Open-answer questions provided some insight on how those improvements impacted on day-to-day situations; decrease of “aggressiveness toward others,” “discomfort in crowded places,” and “autistic meltdown crises / temper tantrums,” for example, allowed families to have less stressful situations and more relaxed moments of joy at home and in outdoor activities. Ninety-five percent of families of patients using FCE in our study reported improvement in their quality of life, which must be considered by any kind of therapeutical intervention for autism (23). Given the variety of symptoms and comorbidities associated with ASD (86), THC-rich and CBD-rich FCEs may be useful for managing different core symptoms of ASD and its comorbidities. Although impairments in sleep quality, appetite and motor development are not central to ASD diagnosis (i.e., non-core symptoms), they are frequent in the spectrum and their improvements are crucial to the well-being of people with ASD and their families.

We have also included in our outcome survey the category “eating non-foods,” which corresponds to the behavioral condition known as allotriophagy or Pica, characterized by the recurrent ingestion of nonfood substances or objects, lacking nutritional value, in an age incompatible with such behavior, according to the DSM 5 (87). In our sample, 8 out of 20 patients (40%) presented this condition. Although its general prevalence in ASD patients is not well established, it seems to be relatively frequent and regularly neglected as an important comorbidity of the spectrum, even though every so often it may lead to important health problems, such as choking, gastrointestinal obstructions or perforations, parasitic infections and poisoning (17–19, 87–90). ASD-associated Pica is generally refractory to medications, and the most effective treatment involves behavioral modeling by applied behavioral analysis therapy (ABA) (87). There is one case report of a female ASD patient who started engaging in coprophagy while in risperidone treatment, which was completely resolved when the medication was changed for aripiprazole (18).

Our results showed that, out of the 8 ASD patients who present Pica, 1 showed moderate worsening, 2 showed no change, 1 showed moderate improvement and 4 showed considerable improvement after FCE treatment. To the best of our knowledge, this is the first time a cannabinoid-based treatment showed such effectiveness for the treatment of Pica in ASD patients. Pica’s etiology may be associated to nutritional deficiencies, related to metabolic or ingestion issues (difficulties on mastication, for instance), to mental conditions, such as psychosis and impaired emotional regulation or a combination of these factors (17, 19, 87). The frequent association of Pica with the ASD populations may be a consequence of comorbid genetic conditions, intestinal inflammatory processes, or food selectivity, for instance. The improvements observed here may be due, therefore, to improvement of absorptive function by reducing intestinal inflammatory processes (91–98) often associated with ASD, and /or by improving emotional regulation. It cannot be ruled out, however, that the whole cannabis extract can also contribute supplementing nutrients (95, 99, 100) absent in the individual’s normal diet.

On a sidenote, we want to address our decisions regarding some symptom categories and subcategories. It is important to stress out that hyperactivity and attention deficit are not necessarily linked, and may, therefore, respond differently to cannabinoids (101–104). Nonetheless, here they are conjugated in one single category because that combination is frequent among ASD patients (102, 103, 105, 106), and further detailing of the specific effect of cannabinoids in each one could broaden too much the scope of the present study. The categories “abnormal behaviors” and “impaired communication and personal interactions” are important to access the core aspects of ASD. However, they are multifaceted and vary widely in specificity and severity among people in the spectrum. For this reason, we have included these categories in the group of main symptomatic categories, and have created two other outcome groups to analyze the specificities for both these categories.

### 4.2. Individual tailoring of dosage and CBD to THC proportion

Regarding the importance of using whole extracts as opposed to purified phytocannabinoids, it is worth noting that in one of the best controlled studies, published by Aran and colleagues in 2021, the treatment with full-spectrum extract containing both CBD and THC has yielded slightly better results than the mixture of pure CBD and THC in proportion equivalent to the whole extract, even though the difference was not statistically significant (73). Besides, a metanalysis study has shown that the use of full-spectrum, CBD-rich extracts has yielded better clinical results than the use of pure CBD for the treatment of infantile syndromes associated with refractory epilepsy and regressive autism (84). Finally, there is one case report showing significant outcome improvements when CBD was combined with selected terpenes for the behavioral treatment of an adolescent ASD patient (107).

Studies that followed the standard 12 weeks period of clinical evaluation and used more restrictive, homogeneous samples (73, 78), may have been too short to assess specific changes in ASD behavioral symptoms, which depend on slow neurochemical, structural and cognitive adjustments. Hopefully, our real-life study design was able to better capture such changes. These more rigorous and well controlled prospective studies have mostly used a single formulation with a CBD to THC proportion of 20: 1 (72–75). Nevertheless, published studies have altogether used a diverse range of full-spectrum cannabis extracts (72–74, 76–79, 82, 108) with comparable results.

Since these studies also varied considerably in terms of sample heterogeneity, treatment time and outcome evaluating method, it is very difficult to compare the results among them. However, most of them presented some level of improvement in both core ASD symptoms and comorbidities, with a varied range of specific benefits and proportion of patients with positive outcomes. Another trend across the studies was the occurrence of mostly mild and infrequent adverse effects. Therefore, we may say that, altogether, these studies indicate that FCE-based treatment of ASD symptoms has yielded some level of positive results, regardless of sample heterogeneity, outcome evaluating method or proportion of CBD to THC dosages. However, when high THC formulations are used, the frequency of behavioral side-effects is higher (72). Hence, it is reasonable to speculate that, even though some patients may not benefit at all from cannabinoid treatment, there might exist a CBD-to-THC formulation range and cannabinoids dosage better suited to each specific condition in the ASD spectrum. The mostly beneficial results demonstrated here support this hypothesis, since our titration protocol used combinations of extracts with different CBD to THC proportions, resulting in individually tailored CBD to THC proportions. Among the 20 patients enrolled in this study, 15 have used CBD-rich formulations throughout treatment and 3 have started with CBD-rich FCEs, and eventually supplemented it with THC-rich FCEs. Among these 18 patients, only one, who belongs to the first group, showed a negative GOS. On the other hand, among the 2 patients who have used a blend of CBD-rich FCE and THC rich FCEs since the beginning, one (8 m) showed a positive result (GOS = 1), while the other (15f) showed a slightly negative score (GOS = −0,3), which resulted from some worsening in behavioral symptoms. Patient 15f was also the only one who did not follow any kind of parallel support activities like psychotherapy or sports (see Supplementary File S1), which may have had some influence over its GOS score. Among the remaining 19 patients, which displayed predominantly positive GOS scores, the most common activities reported were Inclusive Regular Schooling (60% of cases), Psychotherapy (50% of cases), Occupation Therapy and Speech Therapy (45% of cases each) and Special Schooling and Physical Activities (30% of cases each). When proposing a tailored dose FCE treatment, physicians should also observe for the possible relevance of support activities in potentializing the effects of *Cannabis* treatment.

Regardless of our generally small sample size, our data strongly suggests that the effective range of cannabinoid dosage and CBD to THC proportions in ASD treatment may be wide and flexible, but it is safer to start with CBD-rich FCEs, even though some good results may be obtained by starting with a blend of CBD-rich and THC-rich FCEs. Thus, our results allow us to propose safe guidelines for a dosage scheme protocol, as illustrated in Figure 1: (1) low doses of CBD-rich FCEs should be employed at first for all patients as a standard care; (2) CBD doses should be slowly increased until improvements are observed; (3) if improvements are not observed and/or side-effects begin to occur, return to the dosage that has shown no side effects and start supplementing the first CBD-rich FCE with a second THC-rich FCE, gradually increasing in the final proportion of THC to CBD. Of course, these guidelines will need to be further tested with larger and more diverse sample sizes in order to be fully validated.

Cannabinoids are considered potential treatment option for various neurodevelopmental disorders, such as schizophrenia (109–111) and autism. ASD patients have been shown to possess abnormal neuronal activation and connectivity (61, 112, 113), probably due to deficient regulation in synapses. This is a feature reminiscent of mechanisms involved in epilepsy (61). Further, there is higher incidence of epilepsy among autism patients (114, 115) and EEG records of epileptiform activity have been recorded from non-epileptic ASD children (116). Epilepsy symptoms have been successfully treated using Cannabis extracts, especially due to CBD and THC’s ability to reduce epileptiform activity (117–119), reinforcing the use of both as a possible treatment for ASD. Indeed, people on the spectrum present modified expression of cannabinoid receptors (120) and of the endocannabinoid anandamide (121), just like in animal models of autism (122). CBD might further promote ASD symptom improvement through its anti-inflammatory effects (123), as ASD symptoms has been linked to neuroinflammation (124–126). This research background guided our choice of full spectrum Cannabis extracts instead of purified ones, and we believe it plays a part on achieving the benefits reported above.

### 4.3. Side effects and polypharmacy

The specific side effects observed in both patients who were receiving multiple medications and patients who only had used FCE varied considerably, but were mostly mild. Patients 15f and 2 m were the two with the lowest general outcome scores (GOS equal to −0.3 and − 1.1, respectively). Both were using many neuropsychiatric medications and perceived worsening in more symptomatic categories and subcategories than all other patients. The patient with the lowest GOS of all, 2 m, has unilaterally decided to completely withdraw at once both FCE treatment and all other medications after presenting behavioral worsening. He is the only one among all patients who has discontinued FCE treatment in consequence of worsening. He is also the only one, among the six patients who discontinued all previously used medications, who presented negative GOS. In his case, the precocious, unadvised discontinuation of all medications at once precluded us from trying to adjust the doses and have a clear understand of what may have caused the worsening. Medication interactions did not seem to have caused problems in other patients, but it is a possible cause of worsening in this case. Most of the other five patients who discontinued all other medications under clinical supervision did not present negative outcome scores in any of the 10 main symptomatic categories. The only exception was patient 17f, who presented worsening in only one main symptom category (“avoidance and/or restrictions of food intake”), but still had a relatively high outcome score (GOS equal to 1.4).

All the four patients who were not using any other medications since the beginning of the FCE treatment (namely 1 m, 4 m, 9 m and 11 m) presented GOS equal or above 1.0. Concomitant use of FCE and other psychiatric medications was mostly well tolerated across participants, supporting the use of FCE both as an adjuvant and as a single pharmacological approach for ASD treatment. There is, however, significant concern about the important side effects related to polypharmacy of psychotropic medications used by people in the spectrum (26, 27, 30, 34, 35, 38, 41, 42, 44–46, 127–139), prevalent in 19% in adults (138) going as high as 81% in children (136, 137). This may reflect the considerable range of non-core ASD symptoms still untreated by conventional clinical protocols for ASD (86).

Discontinuation and/or dose reduction of other medications, as we observed in a considerable number of cases in this work, may significantly reduce the patient’s array of side effects associated to other medicines (26, 27, 30, 34, 35, 38, 41, 42, 44–46, 127, 129–135, 139). Furthermore, in the long run, such a wide range of benefits may also significantly alleviate the family’s economic burden (22).

### 4.4. Limitations

Although the proposed FCE dosage regimen was developed as a result of clinical experience with over a hundred ASD patients, few patients effectively provided all data necessary to be included in the analysis. As a retrospective study, our cohort is also a convenience sample. The cohort we analyzed is composed of patients who already possessed an ASD diagnosis, often referred from other doctors. As the original evaluation of symptoms and diagnosis was obtained, in some cases, years before the start of Cannabis treatment, records of it were difficult to obtain. The clinicians involved in this study evaluated each person according to DSM-V criteria to perform adequate treatment. Furthermore, one must acknowledge that a patient-reported outcome survey, although valid as a source of clinical information, is not the most objective tool to inform about aspects such as cognitive and motor improvement. Finally, as an open-labeled study with no control group, a possible wishful thinking effect on parents’ answers to the outcome survey must be taken in account, especially in cases of short time treatment and in those were there was no change in previously used medication.

## 5. Conclusion

In sum, this work reinforces the benefits of the full spectrum *Cannabis* extract for treatment of people in the autistic spectrum, and proposes individually-tailored, response-based dosage regimen guidelines for this population. Both the patient’s and their family’s quality of life improved significantly after treatment. Our study expands the scientific data demonstrating that clinical use of *Cannabis* extracts is a safe intervention with promising and valuable effects over many core and comorbid aspects of autism, that are not achieved by conventional medications. In addition, we have shown, for the first time, that allotriophagy (Pica), another important comorbidity relatively frequent in ASD, may also be effectively treated by Cannabis extracts. Side effects from FCE were mild and mostly did not preclude treatment. Further studies with larger samples will be necessary to confirm our overall positive results as well as to further validate our suggested dosage guidelines and new patient/parents-reported outcome questionnaire for ASD.

## Data availability statement

The raw data supporting the conclusions of this article will be made available by the authors, without undue reservation.

## Ethics statement

The studies involving human participants were reviewed and approved by Ethics Committee on Human Research of the Health Sciences College of the University of Brasilia (Universidade de Brasília-UnB). Written informed consent to participate in this study was provided by the participants’ legal guardian/next of kin.

## Author contributions

PM, WM, LS, FC, and RM-L were responsible for study conception and design. PM and LS were responsible for clinical care of the patients. PM, WM, LS, CB, and RM-L were responsible for the elaboration of the patients, parents-reported outcome survey. FC and JB-N provided editing of patients, parents-reported outcome survey. FC was responsible for the ethical aspects of research. WM was responsible for data collection. FC and WM were responsible for data analysis and presentation. PM and RM-L were responsible for the guideline’s graphic presentation. WM and RM-L provided bibliographic revision and wrote the manuscript. JB-N and VB provided critical reading of the manuscript. PM, WM, FC, JB-N, VB, and RM-L provided editing of the manuscript. FC aided in the scientific supervision. RM-L provided general scientific supervision. All authors read and approved the final version of the manuscript.

## Funding

WM received a grant from WeCann Academy, an international institution for education in endocannabinoid medicine, for data collection and analysis in this work. FC was supported Fundação de Apoio ã Pesquisa do Distrito Federal (FAPDF, grant #30023.128.46472.04012022).

## Conflict of interest

PM is one of the founders and main clinicians of NeuroVinci, and founded WeCann Academy, an international institution for education in endocannabinoid medicine. WM received a grant from WeCann Academy for data collection and analysis in this work. RM-L has a 0,8% share of the company Grüne Labs, Pando, Uruguay, which plans to commercialize cannabis-based medications in the future.

The remaining authors declare that the research was conducted in the absence of any commercial or financial relationships that could be construed as a potential conflict of interest.

## Publisher’s note

All claims expressed in this article are solely those of the authors and do not necessarily represent those of their affiliated organizations, or those of the publisher, the editors and the reviewers. Any product that may be evaluated in this article, or claim that may be made by its manufacturer, is not guaranteed or endorsed by the publisher.
