# Supplementary material for: Individually tailored dosage regimen of full-spectrum Cannabis extracts for autistic core and comorbid symptoms: a real-life report of multi-symptomatic benefits
Source: Front Psychiatry. 2023 Aug 21;14:1210155. doi: 10.3389/fpsyt.2023.1210155 (PMC10475955; doi:10.3389/fpsyt.2023.1210155)
Supplement: Supplementary file 1 [file Data_Sheet_1.PDF]

General Data

SURVEY RELEVANCE:

First of all, THANK YOU for taking the time to answer this questionnaire. Autism Spectrum Disorder (ASD) cases are widely varied, just like the composition of the many Cannabis extracts available. So, information about the effects of the Cannabis extract on your child's treatment is important for us. Your answers can help improve Cannabis treatments for your child and for other ASD patients. This research can also help other people to access this treatment. Your answers to the questions ahead will be a huge contribution!

PEOPLE WHO SHOULD ANSWER THE SURVEY:

Ideally, the survey should be answered by the person who lives with the patient and spends most of the time with them (any of the parents or a caregiver) to get the most accurate information. Your answers on the effects of the Cannabis treatment will provide data that we will use in future scientific publications.

.

GUIDELINES FOR FILLING THE SURVEY:

We estimate it takes around 15 minutes for you to finish this survey but it must be answered carefully, so feel free to take breaks between questions or sections. Answers are recorded automatically so you can close the page and pick up from where you stopped at any moment. However, we kindly ask you to finish filling this survey in a week after you begin, at the most. If you have any questions about this questionnaire, you can reach us through email (contato@neurovinci.com.br) or Whatsapp ((48) 98813-2000).

Thank you for your participation!

*Obrigatório

1. Name of the patient *

____

2. Name of the person answering the survey: *

____

3. Relationship with the patient *

Marcar apenas uma oval.

- ( ) Father
- ( ) Mother
- ( ) Brother/Sister
- ( ) Main caregiver
- ( ) Prescribing doctor
- ( ) Patient themselves
- ( ) Outro: ______________________

4. Are you the person who spends most time daily with the patient? *

Marcar apenas uma oval.

- ( ) Yes
- ( ) No

5. Your phone *

____

6. Your email address *

____

7. Which is the level of autism spectrum disorder presented by the patient? *

Marcar apenas uma oval.

- ( ) Level 1 (mild degree of autism)
- ( ) Level 2 (moderate degree of autism)
- ( ) Level 3 (severe degree of autism)

8. What is the patient's degree of verbal communication? *

Marque todas que se aplicam.

- [ ] The patient communicates well using speech.
- [ ] The patient speaks words with communicative intention, but vocabulary is poor and cannot create sentences easily.
- [ ] The patient speaks some words that do not have communicative intention.
- [ ] The patient is non-verbal.
- [ ] The patient emits sounds with communicative intention, but cannot articulate words.
- [ ] The patient communicates well through writing.
- [ ] The patient communicates well through writing, but vocabulary is poor and they struggle to form sentences
- [ ] The patient communicates through alternative means (gestures, cards, apps, others).
- [ ] Outro: ______________________

9. Has the patient been also diagnosed with epilepsy? *

Marcar apenas uma oval.

- ( ) Yes
- ( ) No

10. If the previous answer was "yes", please provide the kind of epilepsy or the associated epileptic syndrome.

____
____
____
____
____

11. Has the patient presented seizures in the 12 months previous to the Cannabis treatment? *

Marcar apenas uma oval.

- Yes
- No

12. Which doctor started and managed the treatment using Cannabis extract? *

Marcar apenas uma oval.

- Dr. Leandro
- Dr. Patrícia

13. When did the Cannabis treatment with either Dr. Patrícia or Dr. Leandro start? *

Exemplo: 7 de janeiro de 2019

14. Was the treatment with Dr. Patrícia or Dr. Leandro interrupted? *

Marcar apenas uma oval.

- Yes
- No

15. If the treatment was interrupted, please inform the approximate date of interruption.

Exemplo: 7 de janeiro de 2019

16. If the treatment was interrupted, please inform the reason for interruption.

Marcar apenas uma oval.

- Previous symptoms worsened.
- There were impactful side effects.
- Treatment was too expensive to maintain.
- Outro: ______________________

17. Inform the biological sex of the patient. *

Marcar apenas uma oval.

- Female
- Male

18. Approximate weight of the patient at onset of treatment: *

____

19. Current weight of the patient or weight of patient at the treatment interruption: *

____

20. Please select the Cannabis extract used during treatment. *

Marcar apenas uma oval.

- ( ) Cannabis oil rich in CBD AMA+ME 1500mg
- ( ) Cannabis oil rich in THC AMA+ME 900mg (10%)
- ( ) Cannabis oil rich in THC AMA+ME 600mg (5%)
- ( ) Cannabis oil rich in CBD MALELI 3000mg (20%)
- ( ) Cannabis oil rich in CBD MALELI 1500mg (10%)
- ( ) Cannabis oil rich in THC MALELI 600mg (5%)
- ( ) Cannabis oil rich in THC MALELI 900mg (10%)
- ( ) Outro: ______________________

21. Dose at the onset (if the patient used more than one type of extract, please specify the doses taken for each) *

____

22. Current dose (or last dose used) *

____

23. School, support therapies and other activities done during treatment. *

Marque todas que se aplicam.

- [ ] Does not attend school yet
- [ ] Inclusive regular school
- [ ] Special school
- [ ] Physiotherapy
- [ ] Occupational therapy
- [ ] Speech therapy
- [ ] Psychotherapy
- [ ] Sports' classes
- [ ] Ecotherapy
- [ ] Communal activities (choirs, art schools, etc)
- [ ] Outro: ______________________

24. Had the patient used any kind of Cannabis-derived product before treatment with Dr. Patrícia or Dr. Leandro? *

Marcar apenas uma oval.

- ( ) Yes
- ( ) No

25. If the previous answer was "yes", please specify what was used, how it was used and for how long.

____
____
____
____
____

Pular para a seção 2 (Evaluation of the Cannabis extract treatment results in autism patients)

Evaluation of the Cannabis extract treatment results in autism patients

GUIDELINES FOR ANSWERING SURVEY SESSIONS 3-17:

The next questions will measure the Cannabis treatment results in 10 separate groups of autism-related symptoms. There are also 5 other groups of treatment impacts on the life and health of the patient and their family. All the 15 groups are as follows:

1) Attention deficit and hyperactivity disorder;
2) Abnormal behaviors;
3) Sadness, melancholy, bad moods;
4) Impaired motor development and motor coordination;
5) Lack of independence for daily activities;
6) Impaired communication and personal interactions (verbal and non-verbal);
7) Intellectual and cognitive deficits;
8) Sleep issues;
9) Seizures;
10) Avoidance and/or restrictions to food intake;
11) Positive mood states;
12) Overall patient's quality of life;
13) Adverse effects due to treatment;
14) Use of other medications;
15) Overall family's quality of life

In general, your answers will consist of choosing one among five options describing the effect of the Cannabis extract over the symptom referred in that session. There are some questions where you will have space to freely describe some aspects.

Feel free to reach out if you have any doubts or questions!

Thank you for helping us improve the quality of life of ASD patients with your answers!

NeuroVinci + University of Brasilia(UnB) research team

Pular para a pergunta 26

1) Attention deficit and hyperactivity disorder

Objective: to measure frequency and intensity of restlessness and excessive moving during everyday activities. Lack of focus. Struggles to remain seated or still during tasks.

26. Please describe in detail and in your own words how did this symptoms present before treatment and how they changed after treatment started. *

27. In your opinion, what was the overall effect of the treatment over these symptoms? *

Marcar apenas uma oval.

- The patient NEVER PRESENTED hyperactivity or attention deficit.
- Symptoms WORSENED SIGNIFICANTLY .
- Symptoms WORSENED MODERATELY.
- Symptoms DID NOT CHANGE.
- Symptoms IMPROVED MODERATELY.
- Symptoms IMPROVED SIGNIFICANTLY.

2) Abnormal behaviors

Objective: to measure frequency and intensity of abnormal behaviors' episodes, such as agression towards others, biting or hitting themselves, repetitive behaviors or movements, excessive apetite or emotional reactions (panic or crying fits, exaggerated laughter), besides other uncommon behaviors like excessive organizing, repeatedly opening and closing doors, unprompted screaming, eating off the floor, eating unedibles (paper, rubbers, etc.), repeating meaningless sounds, etc.

28. Please describe in detail and in your own words how did this symptoms present before treatment and how they changed after treatment started. *

29. In your opinion, what was the overall effect of the treatment over these symptoms? *

Marcar apenas uma oval.

- The patient NEVER PRESENTED those symptoms.
- Symptoms WORSENED SIGNIFICANTLY .
- Symptoms WORSENED MODERATELY.
- Symptoms DID NOT CHANGE.
- Symptoms IMPROVED MODERATELY.
- Symptoms IMPROVED SIGNIFICANTLY.

30. In your opinion, what was the overall effect of the treatment over STEREOTYPIES (shaking hands, twirling in the same spot, rocking back and forth, touching the ears nervously...)? *

Marcar apenas uma oval.

- The patient NEVER PRESENTED those symptoms.
- Symptoms WORSENED SIGNIFICANTLY .
- Symptoms WORSENED MODERATELY.
- Symptoms DID NOT CHANGE.
- Symptoms IMPROVED MODERATELY.
- Symptoms IMPROVED SIGNIFICANTLY.

31. In your opinion, what was the effect of the treatment over AGRESSIVINESS TOWARDS OTHERS? *

Marcar apenas uma oval.

- The patient NEVER PRESENTED those symptoms.
- Symptoms WORSENED SIGNIFICANTLY .
- Symptoms WORSENED MODERATELY.
- Symptoms DID NOT CHANGE.
- Symptoms IMPROVED MODERATELY.
- Symptoms IMPROVED SIGNIFICANTLY.

32. In your opinion, what was the effect of the treatment over SELF-AGRESSIVINESS (self-hitting, self-biting, hitting own head against the wall...)? *

Marcar apenas uma oval.

- The patient NEVER PRESENTED those symptoms.
- Symptoms WORSENED SIGNIFICANTLY.
- Symptoms WORSENED MODERATELY.
- Symptoms DID NOT CHANGE.
- Symptoms IMPROVED MODERATELY.
- Symptoms IMPROVED SIGNIFICANTLY.

33. In your opinion, what was the effect of the treatment over AUTISTIC MELTDOWN CRISIS/TEMPER TANTRUMS? *

Marcar apenas uma oval.

- The patient NEVER PRESENTED those symptoms.
- Symptoms WORSENED SIGNIFICANTLY .
- Symptoms WORSENED MODERATELY.
- Symptoms DID NOT CHANGE.
- Symptoms IMPROVED MODERATELY.
- Symptoms IMPROVED SIGNIFICANTLY.

34. In your opinion, what was the effect of the treatment over SCREAMS AND RANDOM SOUNDS? *

Marcar apenas uma oval.

- The patient NEVER PRESENTED those symptoms.
- Symptoms WORSENED SIGNIFICANTLY .
- Symptoms WORSENED MODERATELY.
- Symptoms DID NOT CHANGE.
- Symptoms IMPROVED MODERATELY.
- Symptoms IMPROVED SIGNIFICANTLY.

35. In your opinion, what was the effect of the treatment over OBSESSIVE COMPULSIVE BEHAVIORS (open and close doors repeatedly, flushing the toilet repeatedly...)? *

Marcar apenas uma oval.

- The patient NEVER PRESENTED those symptoms.
- Symptoms WORSENED SIGNIFICANTLY .
- Symptoms WORSENED MODERATELY.
- Symptoms DID NOT CHANGE.
- Symptoms IMPROVED MODERATELY.
- Symptoms IMPROVED SIGNIFICANTLY.

36. In your opinion, what was the effect of the treatment over EATING NON-FOODS? *

Marcar apenas uma oval.

- The patient NEVER PRESENTED those symptoms.
- Symptoms WORSENED SIGNIFICANTLY .
- Symptoms WORSENED MODERATELY.
- Symptoms DID NOT CHANGE.
- Symptoms IMPROVED MODERATELY.
- Symptoms IMPROVED SIGNIFICANTLY.

37. In your opinion, what was the effect of the treatment over DISCOMFORT IN NOISY OR CROWDED SITUATIONS? *

Marcar apenas uma oval.

- The patient NEVER PRESENTED those symptoms.
- Symptoms WORSENED SIGNIFICANTLY .
- Symptoms WORSENED MODERATELY.
- Symptoms DID NOT CHANGE.
- Symptoms IMPROVED MODERATELY.
- Symptoms IMPROVED SIGNIFICANTLY.

38. In your opinion, what was the effect of the treatment over EXCESSIVE APETITE? *

Marcar apenas uma oval.

- The patient NEVER PRESENTED those symptoms.
- Symptoms WORSENED SIGNIFICANTLY .
- Symptoms WORSENED MODERATELY.
- Symptoms DID NOT CHANGE.
- Symptoms IMPROVED MODERATELY.
- Symptoms IMPROVED SIGNIFICANTLY.

3) Sadness, melancholy, bad moods ;

Objective: to measure frequency and intensity of moments when the patient shows negative emoticons such as sadness, dissatisfaction, annoyance (including crying fits and being upset).

39. Please describe in detail and in your own words how did this symptoms present before treatment and how they changed after treatment started. *

40. In your opinion, what was the overall effect of the treatment over these symptoms? *

Marcar apenas uma oval.

- The patient NEVER PRESENTED those symptoms.
- Symptoms WORSENED SIGNIFICANTLY .
- Symptoms WORSENED MODERATELY.
- Symptoms DID NOT CHANGE.
- Symptoms IMPROVED MODERATELY.
- Symptoms IMPROVED SIGNIFICANTLY.

4) Impaired motor development and motor coordination

Objective: to measure motor troubles during delicate movements, like picking up a pencil, using cutlery or buttoning up a shirt, as well as in daily physical activities (walking, running, exercising, etc).

41. Please describe in detail and in your own words how did this symptoms present before treatment and how they changed after treatment started. *

42. In your opinion, what was the overall effect of the treatment over these symptoms? *

Marcar apenas uma oval.

- The patient NEVER PRESENTED those symptoms.
- Symptoms WORSENED SIGNIFICANTLY .
- Symptoms WORSENED MODERATELY.
- Symptoms DID NOT CHANGE.
- Symptoms IMPROVED MODERATELY.
- Symptoms IMPROVED SIGNIFICANTLY.

5) Lack of independence for daily activities

Objective: to measure the amount of external help needed for eating, dressing up, personal hygiene (using the toilet, showering, etc.), moving around and perfoming other activities.

43. Please describe in detail and in your own words how did this symptoms present before treatment and how they changed after treatment started. *

44. In your opinion, what was the overall effect of the treatment over these symptoms? *

Marcar apenas uma oval.

- The patient NEVER PRESENTED those symptoms.
- Symptoms WORSENED SIGNIFICANTLY .
- Symptoms WORSENED MODERATELY.
- Symptoms DID NOT CHANGE.
- Symptoms IMPROVED MODERATELY.
- Symptoms IMPROVED SIGNIFICANTLY.

6) Impaired communication and personal interactions (verbal and non-verbal);

Objective: to measure the level of communication (in speech, writing, gesturing, using images or apps) and comprehension (in school, in family, among friends, in social media, etc.) difficulties. To measure the lack of visual contact and other difficulties in social interactions.

45. Please describe in detail and in your own words how did this symptoms present before treatment and how they changed after treatment started. *

46. In your opinion, what was the overall effect of the treatment over these symptoms? *

Marcar apenas uma oval.

- The patient NEVER PRESENTED those symptoms.
- Symptoms WORSENED SIGNIFICANTLY .
- Symptoms WORSENED MODERATELY.
- Symptoms DID NOT CHANGE.
- Symptoms IMPROVED MODERATELY.
- Symptoms IMPROVED SIGNIFICANTLY.

47. Concerning verbal communication: *

Marcar apenas uma oval.

- The patient NEVER PRESENTED those symptoms.
- Symptoms WORSENED SIGNIFICANTLY .
- Symptoms WORSENED MODERATELY.
- Symptoms DID NOT CHANGE.
- Symptoms IMPROVED MODERATELY.
- Symptoms IMPROVED SIGNIFICANTLY.

48. Concerning impaired visual contact: *

Marcar apenas uma oval.

- The patient NEVER HAD ISSUES maintaning eye contact.
- Eye contact was SIGNIFICANTLY REDUCED.
- Eye contact was MODERATELY REDUCED.
- Eye contact DID NOT CHANGE.
- Eye contact was MODERATELY IMPROVED.
- Eye contact was SIGNIFICANTLY IMPROVED.

49. Concerning impaired response to their own name *

Marcar apenas uma oval.

- The patient NEVER HAD ISSUES answering when called by their name
- The patient has answered SIGNIFICANTLY LESS when called by their name.
- The patient has answered MODERATELY LESS when called by their name.
- There was no change in answering to their name.
- The patient has answered MODERATELY MORE when called by their name.
- The patient has answered SIGNIFICANTLY MORE when called by their name.

50. Concerning Impaired Attention to Receptive Direct Verbal Communication; *

Marcar apenas uma oval.

- The patient pays MUCH LESS attention when talked to.
- The patient pays SOMEWHAT LESS attention when talked to.
- There was no change in paying attention when talked to.
- The patient pays SOMEWHAT MORE attention when talked to.
- The patient pays MUCH MORE attention when talked to.

51. Concerning Production of Sounds or Isolated Words with Communicative Function *

Marcar apenas uma oval.

- The patient uses MANY LESS sounds or words.
- The patient uses SOMEWHAT LESS sounds or words.
- There was no change in using sounds or words when they are talked to.
- The patient uses SOMEWHAT MORE sounds or words.
- The patient uses MANY MORE sounds or words.

52. Concerning Impaired Written Communication; *

Marcar apenas uma oval.

- The patient has NEVER used writing to communicate.
- Writing has been MUCH LESS used to communicate
- Writing has been SOMEWHAT LESS used to communicate.
- There was no change in using writing for communication.
- Writing has been SOMEWHAT MORE used to communicate.
- Writing has been MUCH MORE used to communicate.

53. Concerning the use of alternative types of communication (such as gestures, signaling, cards, apps and other image-based systems): *

Marcar apenas uma oval.

- The patient has never used alternative types of communication.
- The patient has used MUCH LESS of alternative types of communication.
- The patient has used SOMEWHAT LESS of alternative types of communication.
- There was no change in the use of alternative types of communication.
- The patient has used SOMEWHAT MORE of alternative types of communication.
- The patient has used MUCH MORE of alternative types of communication.

7) Intellectual and cognitive deficits

Objective: to measure the overall difficulty for learning and development at reading, writing, mathematics, arts, use of technology, etc.

54. Please describe in detail and in your own words how did this symptoms present before treatment and how they changed after treatment started. *

55. In your opinion, what was the overall effect of the treatment over these symptoms? *

Marcar apenas uma oval.

- The patient NEVER PRESENTED those symptoms.
- Symptoms WORSENED SIGNIFICANTLY .
- Symptoms WORSENED MODERATELY.
- Symptoms DID NOT CHANGE.
- Symptoms IMPROVED MODERATELY.
- Symptoms IMPROVED SIGNIFICANTLY.

8) Sleep issues

Objective: to measure frequency and intensity of insomnia episodes, trouble sleeping, nocturnal restlessness and waking up in the middle of the night.

56. Please describe in detail and in your own words how did this symptoms present before treatment and how they changed after treatment started. *

57. In your opinion, what was the overall effect of the treatment over these symptoms? *

Marcar apenas uma oval.

- The patient NEVER PRESENTED those symptoms.
- Symptoms WORSENED SIGNIFICANTLY .
- Symptoms WORSENED MODERATELY.
- Symptoms DID NOT CHANGE.
- Symptoms IMPROVED MODERATELY.
- Symptoms IMPROVED SIGNIFICANTLY.

9) Seizures

Objective: to measure frequency and intensity of epileptic seizures (inform type, frequency and duration of seizures).

58. Please describe in detail and in your own words how did this symptoms present before treatment and how they changed after treatment started. *

59. In your opinion, what was the overall effect of the treatment over these symptoms? *

Marcar apenas uma oval.

- The patient NEVER PRESENTED those symptoms.
- Symptoms WORSENED SIGNIFICANTLY .
- Symptoms WORSENED MODERATELY.
- Symptoms DID NOT CHANGE.
- Symptoms IMPROVED MODERATELY.
- Symptoms IMPROVED SIGNIFICANTLY.

10) Avoidance and/or restrictions to food intake;

Objective: to measure the impact of the treatment over moments when they refuse to eat any specific type of food, restraining his diet. To put simply: to measure how heavily the patient refuses to eat fruits, vegetables, meat, grains, dairy food, etc.

60. Please describe in detail and in your own words how did this symptoms present before treatment and how they changed after treatment started. *

61. In your opinion, what was the overall effect of the treatment over these symptoms? *

Marcar apenas uma oval.

- The patient NEVER PRESENTED those symptoms.
- Symptoms WORSENED SIGNIFICANTLY .
- Symptoms WORSENED MODERATELY.
- Symptoms DID NOT CHANGE.
- Symptoms IMPROVED MODERATELY.
- Symptoms IMPROVED SIGNIFICANTLY.

62. Does the patient present any of the conditions below? If they possesses a food allergy not listed please add it in the "Others" section. *

Marque todas que se aplicam.

- [ ] Celiac disease
- [ ] Lactose intolerance
- [ ] Outro: ______________________

11) Positive mood states

Objective: to measure the frequency and intensity of moments when the patient seemed happy, cheerful and in good-spirits, including moments of laughter, smiling and showing affection, seeming satisfied and/or pleased. It is not important here if any of these behaviors were appropriate for the moment they happened, only their frequency and intensity.

63. Please describe in detail and in your own words how were these mood states before treatment and how they changed after treatment started. *

64. In your opinion, what was the overall effect of the treatment over these mood states? *

Marcar apenas uma oval.

- The patient NEVER PRESENTED issues with mood states.
- Symptoms WORSENED SIGNIFICANTLY .
- Symptoms WORSENED MODERATELY.
- Symptoms DID NOT CHANGE.
- Symptoms IMPROVED MODERATELY.
- Symptoms IMPROVED SIGNIFICANTLY.

12) Overall patient's life quality

Objective: to measure the patient's feeling of well-being and quality of life, before and after treatment.

65. Please describe in detail and in your own words how was the patient's quality of life before treatment and how it changed after treatment started. *

66. In your opinion, what was the overall effect of the treatment over the patient's quality of life? *

Marcar apenas uma oval.

- The patient NEVER PRESENTED issues with quality of life.
- The patient's quality of life WORSENED SIGNIFICANTLY .
- The patient's quality of life WORSENED MODERATELY.
- The patient's quality of life DID NOT CHANGE.
- The patient's quality of life IMPROVED MODERATELY.
- The patient's quality of life IMPROVED SIGNIFICANTLY.

13) Adverse effects observed during to treatment

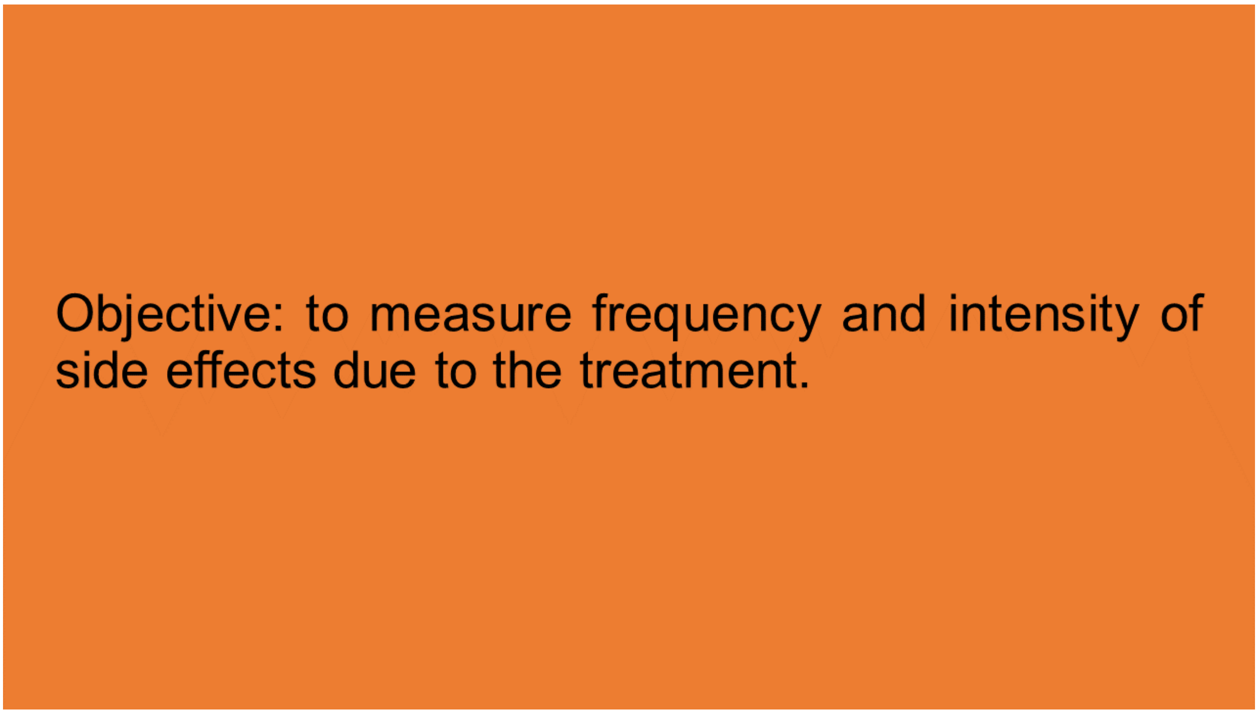

67. Were there any side effects due to the use of Cannabis extract? If there were, please describe which were they, their frequency, intensity and duration, in your own words. *

68. Did the treatment cause any of the side effects below? *

Marque todas que se aplicam.

	Did not occur	Mild	Moderate	Severe	Only in the beggining of treatment	Still ongoing
**Lack of sleep**	☐	☐	☐	☐	☐	☐
**Excessive sleepiness**	☐	☐	☐	☐	☐	☐
**Agitation**	☐	☐	☐	☐	☐	☐
**Apathy**	☐	☐	☐	☐	☐	☐
**Diarrhea**	☐	☐	☐	☐	☐	☐
**Constipation**	☐	☐	☐	☐	☐	☐
**Vomiting**	☐	☐	☐	☐	☐	☐
**Fever**	☐	☐	☐	☐	☐	☐
**Seizures**	☐	☐	☐	☐	☐	☐
**Eye redness**	☐	☐	☐	☐	☐	☐
**Urinary incontinence**	☐	☐	☐	☐	☐	☐
**Abnormal thirst**	☐	☐	☐	☐	☐	☐
**Excessive apetite**	☐	☐	☐	☐	☐	☐
**Lack of apetite**	☐	☐	☐	☐	☐	☐
**Loss of weight**	☐	☐	☐	☐	☐	☐
**Weight gain**	☐	☐	☐	☐	☐	☐
**Allergic reaction**	☐	☐	☐	☐	☐	☐

Others	☐	☐	☐	☐	☐	☐

14) Use of other medications

Objective: to point out previous use of other medications and how it might have changed after starting the Cannabis extract treatment.

69. Does the patient take (or used to take) medicine other than the Cannabis extract? Which were the drugs? Was there any change in dosage after starting the treatment? Was any drug removed or replaced? Can you explain how medicine use changed over the course of the Cannabis treatment? If possible, please describe the changes and their reasons in detail. *

15) Overall family's quality of life

Objective: to measure the feeling of well-being and the quality of life of the patient's family, before and after treatment.

70. Please describe in detail and in your own words how was the family's quality of life before treatment and how it changed after treatment started. *

71. In your opinion, what was the overall effect of the treatment over the family's quality of life? *

Marcar apenas uma oval.

- The patient NEVER PRESENTED issues with quality of life.
- The family's quality of life WORSENED SIGNIFICANTLY .
- The family's quality of life WORSENED MODERATELY.
- The family's quality of life DID NOT CHANGE.
- The family's quality of life IMPROVED MODERATELY.
- The family's quality of life IMPROVED SIGNIFICANTLY.

Este conteúdo não foi criado nem aprovado pelo Google.

Google Formulários
